# Diferenças entre Duas Condições Cardíacas Hipertróficas Distintas: Doença de Fabry Versus Cardiomiopatia Hipertrófica

**DOI:** 10.36660/abc.20230229

**Published:** 2024-01-24

**Authors:** Onur Akhan, Mehmet Kış, Tuncay Güzel, Mehdi Zoghi

**Affiliations:** 1 Bilecik Training and Research Hospital Bilecik Turquia Bilecik Training and Research Hospital – Cardiology, Bilecik – Turquia; 2 Dokuz Eylul University Faculty of Medicine Izmir Turquia Dokuz Eylul University Faculty of Medicine – Cardiology, Izmir – Turquia; 3 Health Science University Gazi Yasargil Training and Research Hospital Diyarbakır Turquia Health Science University , Gazi Yasargil Training and Research Hospital – Cardiology, Diyarbakır – Turquia; 4 Ege University Faculty of Medicine Izmir Turquia Ege University Faculty of Medicine – Cardiology, Izmir – Turquia

**Keywords:** Cardiomiopatias, Hipertrofia Ventricular Esquerda, Cardiomiopatia Hipertrófica, Doença de Fabry

## Abstract

**Fundamento:**

A cardiomiopatia hipertrófica (CMH) e a doença de Fabry (DF) são doenças herdadas geneticamente com características fenotípicas de hipertrofia ventricular esquerda (HVE) que causam resultados cardíacos adversos.

**Objetivos:**

Investigar as diferenças demográficas, clínicas, bioquímicas, eletrocardiográficas (ECG) e ecocardiográficas (ECO) entre CMH e DF.

**Métodos:**

60 pacientes com CMH e 40 pacientes com DF foram analisados retrospectivamente como uma subanálise do “estudo LVH-TR” após exclusão de pacientes com fibrilação atrial, ritmo de estimulação, bloqueios de ramo e bloqueios atrioventriculares (AV) de segundo e terceiro graus. O nível de significância foi aceito como <0,05.

**Resultados:**

O sexo masculino (p=0,048) e a creatinina (p=0,010) são significativamente maiores a favor da DF; entretanto, infradesnivelamento do segmento ST (p=0,028), duração do QT (p=0,041), espessura do septo interventricular (SIVd) (p=0,003), espessura da parede posterior (PWd) (p=0,009), insuficiência mitral moderada a grave (IM) (p=0,013) e o índice de massa ventricular esquerda (IMVE) (p=0,041) são significativamente maiores a favor da CMH nas análises univariadas. Na análise multivariada, a significância estatística apenas permanece na creatinina (p=0,018) e na duração do intervalo QT (0,045). A DF foi positivamente correlacionada com a creatinina (rho=0,287, p=0,004) e a CMH foi positivamente correlacionada com o PWd (rho=0,306, p=0,002), IVSd (rho=0,395, p<0,001), IM moderada-grave (rho= 0,276, p<0,005), IMVE (rho=0,300, p=0,002), espessura relativa da parede (ERP) (rho=0,271, p=0,006), duração do QT (rho=0,213, p=0,034) e depressão do segmento ST (rho =0,222, p=0,026).

**Conclusão:**

Características bioquímicas, ECG e ECO específicas podem auxiliar na diferenciação e no diagnóstico precoce da CMH e da DF.

## Introdução

A doença de Fabry (FD) é uma doença de armazenamento lisossômico ligada ao cromossomo X, caracterizada pela deficiência da enzima a-galactosidase A (a-Gal A), que deposita globotriaosilceramida (Gb3) e glicoesfingolipídios relacionados e afeta os sistemas vascular, renal, neurológico e cardíaco. ^1 - 3^ A DF pode ser classificada como clássica (grave) e de início tardio (não clássica - envolvimento limitado dos órgãos). O envolvimento cardíaco é o principal fator prognóstico e é frequentemente caracterizado por aumento da espessura/massa da parede do ventrículo esquerdo (VE), anormalidades funcionais, valvopatia, arritmias e insuficiência cardíaca. ^3 , 4^ A incidência de DF varia de 1/40.000 a 1/117.000, e a prevalência de DF na hipertrofia inexplicável do VE (HVE) varia de 0% a 12%. ^1 , 5 - 10^ Em um estudo realizado em 2023, 19,5% dos pacientes com hipertrofia do VE de origem desconhecida apresentavam diminuição da atividade da enzima a-Gal A. ^11^ Devido à inativação aleatória do cromossomo X, pacientes do sexo feminino podem apresentar sintomas tão graves quanto pacientes do sexo masculino ou ser assintomáticos. ^1 - 4 , 12^ A natureza ligada ao cromossomo X da DF causa disparidades diagnósticas entre os sexos. A avaliação da menor atividade de a-Gal em pacientes do sexo masculino é diagnóstica. A atividade da a-Gal A pode ser limítrofe ou normal em mulheres, portanto o sequenciamento genético às vezes é a única maneira de diagnosticar. ^12 , 13^

A cardiomiopatia hipertrófica (CMH), uma doença cardíaca genética de transmissão autossômica dominante (causada por uma mutação nas proteínas do sarcômero) com características fenotípicas de HVE com uma incidência de 1/500, causa consequências cardíacas graves, como arritmias ventriculares, maior risco de insuficiência cardíaca e morte cardíaca súbita. ^13 - 22^

As diretrizes da CHM recomendam a investigação de causas atípicas de HVE, como a DF. ^5 , 15 , 16^ As mulheres são subdiagnosticadas em ambas as doenças, talvez devido às características da doença ou aos métodos de triagem. ^12 , 17 , 18 , 23^ O reconhecimento e a diferenciação precoces são necessários para tratar a DF e a CMH, especialmente o envolvimento cardíaco. Consequentemente, esses distúrbios podem ser tratados precocemente, melhorando a qualidade de vida dos pacientes. ^3 , 4 , 15 , 16 , 20 - 24^

Em nosso estudo, objetivamos avaliar as características eletrocardiográficas (ECG) e ecocardiográficas (ECO) de forma padronizada, minimizando os possíveis fatores de confusão com exclusão de fibrilação atrial (FA), Bloqueio de ramo direito (BRD), bloqueios atrioventriculares (AV) de 2º e 3º graus e o ritmo do marca-passo para o início diagnóstico e diferenciação de ambas as doenças.

## Métodos

Nosso estudo é uma análise de subgrupo do estudo nacional, multicêntrico, observacional e de triagem ‘LVH-TR’ realizada em 22 centros entre fevereiro de 2020 e agosto de 2021. No estudo LVH-TR, a taxa de pacientes com diagnóstico de CMH foi de 7,5% (66 pacientes). Na avaliação inicial do estudo LVH-TR, fatores como hipertensão, valvulopatias, doenças cardíacas congênitas, insuficiência renal crônica, cardiomiopatias infiltrativas, coração de atleta e cardiomiopatia ventricular esquerda não compactada foram reconhecidos como possíveis causas de HVE. Os pacientes que foram avaliados inicialmente e apresentaram HVE de origem desconhecida foram submetidos ao algoritmo DF. Pacientes com suspeita de DF, com achados clínicos (dor neuropática, dor de estômago, diarreia, hipoidrose,…), exame físico (angioqueratoma, perda auditiva, opacidades da córnea…), laboratoriais (proteinúria…), ECG, ECO e ressonância magnética cardíaca foram considerados para avaliação adicional. Níveis baixos da enzima α-Gal-A foram detectados em 43 pacientes com HVE avaliados no grupo DF do nosso estudo. Os pacientes Fabry em nosso estudo consistiam em fenótipos geralmente mais leves. A mutação do gene GLA foi geralmente vista como uma mutação *missense* e foi positiva em 14 pacientes, dos quais 5 eram do sexo feminino e 9 do sexo masculino. Pacientes do sexo feminino não apresentavam mutações variantes de significado incerto. ^25 , 26^ DF foi diagnosticada com menor atividade de a-Gal em homens e análise de mutação genética em mulheres em pacientes com HVE inexplicada. Embora a menor atividade de A-Gal A em homens seja suficiente para o diagnóstico, a análise genética foi realizada para avaliação adicional. Notavelmente, indivíduos com diagnóstico preexistente de DF não foram incluídos. Assim, se verifica que nenhum dos pacientes identificados com DF recebe terapia de reposição enzimática durante o período diagnóstico. Nosso estudo não observou relação familiar entre os pacientes com diagnóstico de DF.

Os pacientes com bloqueio AV de 2º e 3º graus, BRD, FA e ritmo de marca-passo foram excluídos do estudo para comparação dos parâmetros eletrocardiográficos de forma padronizada. Após a exclusão, foram incluídos no estudo 60 pacientes com CMH e 40 com DF em ritmo sinusal (o diagrama de Consort e o resumo dos resultados são mostrados na Figura Central ).

**Figura Central:**
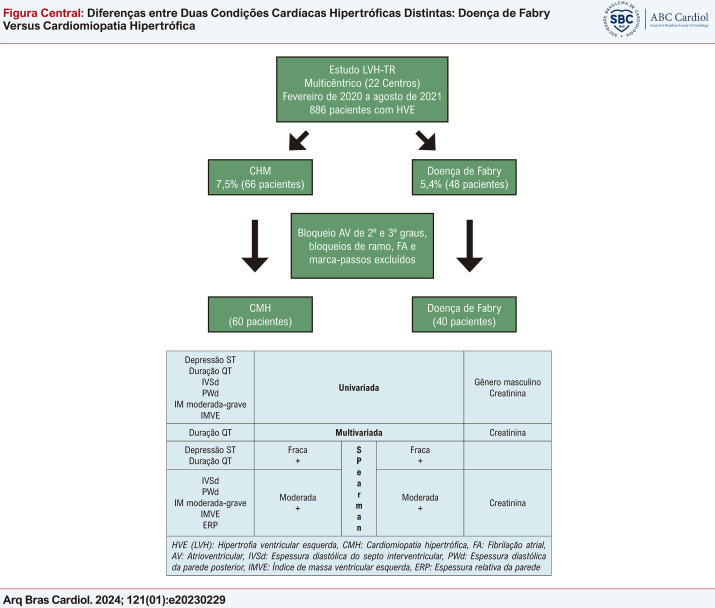
: Diferenças entre Duas Condições Cardíacas Hipertróficas Distintas: Doença de Fabry Versus Cardiomiopatia Hipertrófica Diagrama Consort e resumo dos resultados.

Características demográficas, sintomas, medicamentos, dados de medição bioquímica padrão (glicose no sangue, HgA1c, hemoglobina, ureia, creatina, taxa de filtração glomerular (TFG), troponina T de alta sensibilidade (TnT), Nt-pro-BNP), dados de ECG e ECO foram comparados para CHM e DF.

### Análise eletrocardiográfica

Em repouso, a frequência cardíaca, o tempo PR, a largura do QRS e a duração do QT foram avaliados a partir dos ECGs padrão de registro de 12 derivações (velocidade do papel de 10 mV/mm e 25 mm/s). A fórmula de Bazett (QT/√RR) mede o intervalo QT corrigido (QTc). Além disso, negatividade T, achatamento da onda T, depressão do segmento ST (os valores do limiar de depressão do ponto J são -0,05 mV nas derivações V2 e V3 e -0,1 mV em todas as outras derivações para homens e mulheres) e Índice de Sokolow-Lyon A positividade (SLI = SV1 + VD5 ou V6 ≥ 35 mm) foi registrada de acordo com as recomendações das diretrizes de eletrocardiografia da *American Heart Association* .

### Análise ecocardiográfica

A avaliação ECO transtorácica padrão com eixo longo paraesternal, eixo curto paraesternal, incidências apicais de duas câmaras e apicais de quatro câmaras são avaliadas de acordo com as diretrizes atuais. A HVE é diagnosticada pelo IMVE, corrigido pela área de superfície corporal (ASC). A massa do VE é calculada com a fórmula 0,8×1,04×[(DDVE + IVSd +PWd)3 – DDVE3]+0,6, e o IMVE também é calculado com a fórmula Massa do VE/SC. A espessura relativa da parede (ERP) é calculada pela fórmula (2 x LV-PWd / DDVE), e o modo M é usado para a medição do IVSd na fase diastólica (DDfVE: diâmetro diastólico final do ventrículo esquerdo, IVSd: espessura diastólica do septo interventricular , PWd: Espessura diastólica da parede posterior).

Após a exclusão de doenças sistêmicas, como miopatias mitocondriais, doenças de armazenamento de glicogênio/lisossomas em crianças, DF, amiloidose, sarcoide, hemocromatose e cardiomiopatia de Danon e causas secundárias de HVE, como coração de atleta, cardiomiopatia hipertensiva, doença renal crônica (DRC), obstrução hemodinâmica causada por lesões obstrutivas do lado esquerdo (estenose valvular ou subvalvar), ou obstrução após infarto ântero-apical e cardiomiopatia de estresse, espessura de parede ≥15 mm em um ou mais segmentos miocárdicos no VE é diagnosticada como CMH.

### Teste de atividade enzimática de α-Galactosidase A

Um método fluorimétrico é usado para medir a atividade da enzima α-Gal-A (AGAL), com teste de sangue seco obtido pela aspiração de amostras de sangue venoso periférico em papel de amostra de sangue seco (Substrato: 4-Metilumbeliferil-α-D-galactopiranosídeo (TRC, M334475) - Inibidor: N-Acetil-D-galactosamina (Sigma, A2795)). A incubação foi feita a 37°C durante 17 horas e a reação foi interrompida. A fluorescência foi registrada no fluorímetro (Ex: 366 nm - Em: 442 nm), e a curva de calibração foi criada (4-Metilumbeliferona (Sigma M1381)). O valor limite foi determinado como >2,50 nmol/mL/h para a faixa usual de atividade AGAL pelo teste de característica de operação do receptor (ROC) realizado pelo ‘grupo de laboratório Duzen’.

### Análise de mutação

Em relação à análise genotípica para DF, a análise da sequência do gene GLA foi realizada com o método de sequenciamento de próxima geração (NGS). Os produtos de PCR amplificados a partir do DNA isolado foram sequenciados e comparados com a sequência de referência (NCBI Genomic Reference Sequence: NG 007119.1, NM 000169.2). As mutações da sequência codificante foram relatadas entre as mutações encontradas neste banco de dados. Além disso, foi adicionada a associação de mutações relatadas com DF dos bancos de dados ‘HGMD’ e ‘ClinVar’. Programas de análise de modelo como SIFT, Mutation t@ster e previsões PolyPhen-2 foram adicionados para mutações que não estão no banco de dados.

### Análise estatística

Foi utilizado o programa IBM SPSS Statistics 25.0. A conformidade das variáveis numéricas com a distribuição normal foi examinada pelo teste de Kolmogorov-Smirnov. Para comparar os dois grupos em termos de variáveis numéricas, foi utilizado o teste-t para amostras independentes, caso a distribuição normal fosse alcançada, e o teste U de Mann-Whitney, caso contrário. As variáveis categóricas foram apresentadas como números (n) e proporções (%). A relação entre variáveis categóricas foi examinada com o teste Qui-quadrado de Pearson e Exato de Fisher. O poder da CMH e do valor da DF na predição foi avaliado com análises de regressão logística univariada e multivariada. Os valores de Odd’s Ratio (OR) e IC 95% foram registrados. Os parâmetros preditores de CMH e DF foram avaliados com análise de correlação de Spearman. Os valores de rho e p foram registrados. Os dados descritivos foram expressos como valores de média ± desvio padrão (DP) para variáveis contínuas com distribuição normal e valores de mediana (intervalo interquartil - IIQ) para variáveis com distribuição não normal. O nível de significância para todas as hipóteses foi aceito como <0,05.

## Resultados

Em ambas as doenças, a proporção de pacientes do sexo masculino foi maior. Palpitações, sintomas de tontura e taxa de uso de betabloqueadores foram encontrados em maior proporção na CMH do que na DF. TFG, creatinina e TnT foram significativamente maiores em favor da DF. As comparações entre os grupos em relação às características demográficas, clínicas, uso de medicamentos e parâmetros bioquímicos estão resumidas na Tabela 1 .

**Tabela 1:** – Comparação de características demográficas, clínicas, uso de medicamentos e parâmetros bioquímicos

Parâmetros	CMH (n=60)	Fabry (n=40)	valor p
Idade, média±dp	53,9±14,0	51,9±12,9	0,483
Sexo masculino, n (%)	42 (70,0)	35 (87,5)	0,042
Índice de Massa Corporal, kg/m ^2^ , média±dp	29,0±5,0	27,5±4,2	0,139
Área de superfície corporal, média±dp	1,94±0,19	1,96±0,17	0,655
Pressão Arterial Sistólica, média±dp	132+22	138±23	0,208
Pressão Arterial Diastólica, média ± dp	79±11	84±13	0,055
Hiperlipidemia, n (%)	13 (21,7)	5 (12,5)	0,242
Diabetes Mellitus, n (%)	10 (16,7)	5 (12,5)	0,568
**Sintomas**			
Dor no peito, n (%)	27 (45,0)	16 (40,0)	0,621
Palpitações, n (%)	33 (55,0)	9 (22,5)	0,001
Dispneia, n (%)	34 (56,7)	16 (40,0)	0,102
Cansaço, n (%)	30 (50,0)	18 (45,0)	0,624
Tontura, n(%)	21 (35,0)	6 (15,0)	0,027
Síncope, n (%)	6 (10,0)	2 (5,0)	0,367
**Uso de drogas**			
Bloqueador dos canais de cálcio, n (%)	12 (20,0)	10 (25,0)	0,554
β-bloqueador, n (%)	51 (85,0)	20 (50,0)	<0,001
Amiodarona, n (%)	2 (3,3)	0	0,515*
Estatina, n (%)	11 (18,3)	5 (12,5)	0,436
**Parâmetros Bioquímicos**			
Hb, g/dl, média±dp	13,8±1,6	13,8±2,0	0,852
HbA1c, %, média±dp	5,7±0,7	5,8±0,5	0,705
Creatinina, mg/dl, mediana (intervalo interquartil)	0,90 (0,80-1,00)	1,00 (0,86-1,34)	0,004
TFG (ml/min)	90,7±27,1	73,9±31,0	0,005
Nt±probnp, pg/ml, mediana (intervalo interquartil)	321 (212-481)	192 (156-656)	0,240
Troponina, pg/ml, mediana (intervalo interquartil)	11,5 (5,0-22,7)	44,0 (15,2-125,0)	<0,001

CMH: cardiomiopatia hipertrófica; BCC: bloqueador dos canais de cálcio; BB: betabloqueador; Hb: hemoglobina; TFG: taxa de filtração glomerular; *Teste Exato de Fischer.

As diferenças detectadas nas características do ECG foram que a depressão do segmento ST foi maior e a duração do QT foi maior na CMH do que na DF. A disfunção diastólica do ventrículo esquerdo (DDVE) grau I foi maior a favor da DF, enquanto a DDVE grau III foi maior a favor da CMH. A Tabela 2 mostra todos os parâmetros de ECG e ECO para outras especificidades e diferenças. Em nosso estudo também temos a informação de que o percentual de OVSVE em pacientes com CMH foi de 38,3% e o percentual de MAS foi de 43,3%.

**Tabela 2:** – Comparação de parâmetros eletrocardiográficos e ecocardiográficos

Eletrocardiografia Parâmetros		CMH (n=60)	Fabry (n=40)	valor p
Frequência cardíaca, média ± dp		73±15	73±14	0,883
Bloqueio AV de 1º grau, n (%)		8 (13,3)	6 (15,0)	0,814
Negatividade T, n (%)		39 (65,0)	22 (55,0)	0,315
Achatamento T, n (%)		16 (26,7)	9 (22,5)	0,637
Depressão ST, n (%)		39 (65,0)	17 (42,5)	0,026
Distância PR, msn, média±dp		162,3±31,8	151,5±36,7	0,124
Duração do QRS, msn, média±dp		102,3±12,3	98,0±13,9	0,106
Duração do QT, msn, média±dp		412,7±65,1	388,1±39,1	0,035
Duração do QTc, média±dp		447,3±70,9	423,7±39,1	0,058
Índice Sokolow-Lyon, n (%)		44 (73,3)	24 (60,0)	0,161
**Parâmetros Ecocardiográficos**				
FEVE, média±dp		58,6±7,4	57,9±5,8	0,588
FEVD ≥ 45, n (%)		57 (95,0)	40 (100,0)	0,151
DDVE Classificação	DDVE Grau I	25 (41,7)	24 (60,0)	0,016
	DDVE Grau II	18 (30,0)	8 (20,0)	
	DDVE Grau III	14 (23,3)	2 (5,0)	
DDVE, mm, média±dp		43,2±6,2	44,7±3,7	0,148
DSVE, mm, média±dp		28,8±7,3	27,7±4,0	0,310
IVSd, mm, média±dp		19,5±4,4	16,5±4,1	0,001
PWd, mm, média±dp		15,3±4,0	13,3±2,5	0,002
E lateral, mm, média±dp		8,9±2,9	6,7±1,6	<0,001
E Septal, mm, média±dp		6,3±2,3	5,5±1,6	0,041
Raiz Aórtica, mm, média±dp		27,1±4,5	27,0±4,3	0,898
Seio de Valsalva, mm, média±dp		35,4±4,0	35,5±4,7	0,917
Aorta ascendente, mm, média±dp		34,9±3,7	35,1±3,9	0,848
Diâmetro VD, mm, média±dp		28,9±4,7	27,6±3,9	0,154
TAPSE mm		20,7±3,5	19,7±4,0	0,232
PABs, mmHg		29,8±9,6	23,9±5,6	<0,001
E/e′ média > 14, n (%)		23 (38,3)	18 (45,0)	0,507
Volume do AE, mL/m ^2^ , média±dp		62,7±28,7	53,5±11,3	0,028
Velocidade máxima de TR > 2,8 m/s, n (%)		13 (21,7)	7 (17,5)	0,610
IM moderada-grave, n (%)		16 (26,7)	2 (5,0)	0,006
IMVE, g/m ^2^ , média±dp		161,5±32,0	143,7±50,1	0,033
ERP, média±dp		0,72±0,25	0,60±0,15	0,003

*CMH: cardiomiopatia hipertrófica; AV: atrioventrıcular; FEVE: fração de ejeção do ventrículo esquerdo; FEVD: fração de ejeção do ventrículo direito; DDVE: diâmetro diastólico final do ventrículo esquerdo; DSVE: diâmetro sistólico final do ventrículo esquerdo; IVSd: espessura diastólica do septo interventricular; PWd: espessura da parede posterior diastólica; PABs: pressão sistólica da artéria pulmonar; AE: átrio esquerdo; TR: insuficiência tricúspide; IM: insuficiência mitral, AR: insuficiência aórtica, IMVE: índice de massa ventricular esquerda; VE: ventrículo esquerdo; ERP: espessura relativa da parede; MAS: movimento anterior sistólico.*

Os preditores e preditores independentes foram resumidos na Tabela 3 devido à análise de regressão logística univariada e multivariada. Esses resultados também serão explicados mais detalhadamente na seção de discussão. Na análise de correlação de Spearman, também encontramos uma correlação positiva moderada entre DF e creatinina e uma correlação positiva moderada entre CMH e PWd, IVSd, IM moderada-grave, IMVE, EPR e uma correlação positiva fraca entre CMH e duração do QT e depressão de ST ( Tabela 4 ).

**Tabela 3:** – Análises de regressão univariada - multivariada para determinação de preditor de cardiomiopatia hipertrófica e doença de Fabry

Parâmetros	Univariada Análise		Multivariada Análise	
	OR (IC 95%)	valor p	OR (IC 95%)	valor p
Sexo masculino	0,333 (0,112-0,989)	0,048	0,561 (0,154-2,046)	0,381
Creatinina	7.313 (1.623-32.954)	0,010	6.405 (1.371-29.925)	0,018
Troponina	1,004 (0,999-1,010)	0,130		
Depressão ST	2.513 (1.105-5.712)	0,028	2,025 (0,719-5,702)	0,181
Duração do QT	0,991 (0,983-1,000)	0,041	0,989 (0,978-1,000)	0,045
IVSd	0,836 (0,744-0,940)	0,003	0,945 (0,796-1,121)	0,513
PWd	0,820 (0,707-0,951)	0,009	0,847 (0,695-1,031)	0,098
Volume AE	0,982 (0,963-1,001)	0,063		
IM moderada a grave	6.909 (1.492-31.994)	0,013	4,660 (0,702-30,939)	0,111
IMVE	0,987 (0,975-0,999)	0,041	1,004 (0,985-1,023)	0,692

*OR: odds ratio; IC: intervalo de confiança; IVSd: espessura diastólica do septo interventricular; DP: espessura diastólica da parede posterior, IM: insuficiência mitral, IMVE: índice de massa ventricular esquerda.*

**Tabela 4:** – Análise de Correlação de Spearman para Determinar Preditor de Cardiomiopatia Hipertrófica e Doença de Fabry

	Creatinina	ST Depr.	QT Dur.	IVSd	PWd	IM Mod.-Sev.	IMVE	ERP
rho	0,287	-0,222	-0,213	-0,395	-0,306	-0,276	-0,300	-0,273
valor p	0,004	0,026	0,034	<0,001	0,002	0,005	0,002	0,006

*Depr.: depressão; Dur.: duração; IVSd: espessura diastólica do septo interventricular; PWd: espessura diastólica da parede posterior, Mod.-Sev: moderada-grave; IM: insuficiência mitral; IMVE: índice de massa ventricular esquerda; ERP: espessura relativa da parede.* *+ valores estão correlacionados com a Doença de Fabry / - os valores estão correlacionados com a Cardiomiopatia Hipertrófica.*

## Discussão

Nosso estudo examina características demográficas, clínicas, bioquímicas, de ECG e ECO de DF e CMH. Devido às suas semelhanças e potenciais resultados adversos, estas duas doenças devem ser distinguidas. Assim como a literatura, nossa população de estudo teve mais pacientes do sexo masculino em ambas as doenças. ^1 - 4 , 15 - 18^ As características hereditárias da DF, seu curso assintomático no sexo feminino, possível viés de triagem e modificadores genéticos e hormonais na CMH explicam essa situação. Em nosso estudo, o percentual do sexo masculino também foi significativamente maior no DF do que no CHM. No entanto, Jungua et al. não detectaram diferença significativa no sexo masculino (p=0,42), e no estudo de Sacchari et al., nenhum valor de p foi atribuído em relação à discrepância entre os sexos masculinos. ^5 , 22^ Possíveis variações nas características demográficas podem ter causado essa diferença.

Embora existam algumas diferenças na ordem de frequência, angina, dispneia, palpitações e síncope são os sintomas cardíacos comuns em ambas as doenças. ^15 , 16 , 24^ A síncope também está incluída na pontuação de risco de morte súbita cardíaca do CHM. Os exames de triagem, e não os sintomas, levam ao diagnóstico de CMH, de acordo com as diretrizes. ^15 , 16^ Embora os sintomas comuns em nosso estudo fossem consistentes com as diretrizes, as diferenças entre os grupos foram consideradas coincidentes porque esses sintomas eram subjetivos e inespecíficos.

Betabloqueadores, verapamil e diltiazem diminuem a frequência cardíaca para diminuir as pressões diastólicas do VE e melhorar o enchimento do VE na CMH. Os ß-bloqueadores são inicialmente titulados para a dose máxima tolerável para obstrução sintomática da via de saída do ventrículo esquerdo (OVSVE). ^15 , 16^ Além da reposição enzimática e da terapia com acompanhantes, medicamentos concomitantes também podem ser usados para a redução das queixas, retardando/prevenindo o progressão das manifestações orgânicas e melhora da qualidade de vida na DF. ^27 , 28^ Em nosso estudo, o uso de ß-bloqueadores foi significativamente maior na CMH em comparação com a DF, semelhante ao estudo de Jungua N. et al. (84% vs. 26%, respectivamente; p<0,001). ^5^

O acúmulo gradual de GL3 em todos os tipos de células renais, predominantemente em podócitos, e a liberação de mediadores inflamatórios causam nefropatia por DF. ^29^ A DRC é excluída da CMH devido à definição nas diretrizes atuais. ^15^ Mas a CMH também pode prejudicar a função renal secundária a desfechos cardíacos adversos. ^30^ Em nosso estudo, a creatinina e o TnT são significativamente maiores, e a TFG é significativamente menor na DF do que na CMH (todos os valores de p <0,05). Como o estudo LVH-TR foi um estudo de triagem e, em primeiro lugar, as condições com etiologia clara de HVE foram determinadas e posteriormente avaliadas para a etiologia inexplicada de HVE, e então o diagnóstico de DF foi alcançado, e talvez devido às características da população, o envolvimento renal em pacientes com DF pode ter sido em níveis baixos (TFG: 73,9±31,0 ml/min em DF). ^25^ DF e CMH podem aumentar indicadores de lesão miocárdica como a troponina (especialmente com OVSVE). ^31 - 33^ Foi afirmado que a disfunção endotelial pode ser observada na cardiomiopatia de Fabry (definida como espessura da parede do VE igual ou superior a 12 mm na ressonância magnética cardíaca) devido a alterações nos marcadores de angiogênese. Neste estudo, os valores de TFG foram 95,7 ± 19,6 (ml/min/1,73 m ^2^ ) no grupo sem cardiomiopatia, 71,9 ± 21,5 no grupo com cardiomiopatia e 3,7 ± 0,8 e 28,8 ± 25,2 no TnT (pg/ml), respectivamente. ^31^ Em estudo sobre biomarcadores séricos em pacientes com CMH e FEVE preservada, a diminuição dos parâmetros de *strain* foi apontada como a razão para o aumento dos valores de TnT em relação ao grupo controle saudável. Neste estudo, o valor mediano da troponina T foi de 14,25 pg/ml (IIQ: 9,98, 22,83) em 64 pacientes com CMH, 65% dos quais tinham OVSVE e 10% que tinham FA. ^34^ O aumento nos valores de TnT em Pacientes com CMH têm sido associados a arritmias e estágios da doença. ^35^ Em nosso estudo, os valores de TnT foram semelhantes aos mencionados acima. As diferenças entre os grupos podem ser interpretadas com CMH com relação OVSVE (38%), exclusão de arritmias e possíveis fatores de confusão, como diferenças relacionadas aos parâmetros de função renal.

Em Jungua et al., o BRD (54% vs. 22%, respectivamente; p=0,001), duração do QRS (117±27 msn vs. 99±25, respectivamente; p<0,001) e SLP (p=0,004) foram significativamente maiores no grupo DF do que no grupo CHM. No entanto, nenhuma diferença significativa foi encontrada em outros parâmetros (todos os valores de p<0,05), como QTc (p = 0,58 *)* . ^5^ Em nosso estudo, a CMH apresentou maior duração do QT e depressão do segmento ST (medida não observada em estudos anteriores) do que DF. O SLI positivo foi comparável em ambos os grupos (p=0,161). Na análise univariada, a depressão do segmento ST e a duração do QT predizem a CMH; entretanto, apenas a duração do QT prediz CMH na análise multivariada. A depressão do segmento ST, a duração do QT e a CMH foram correlacionadas positivamente de forma fraca na análise de correlação de Spearman. À medida que a CMH e a DF progridem, o QT pode prolongar-se. ^36 , 37^ Assim, a percentagem de OVSVE da CMH e a DF em fases anteriores podem ter afetado as discrepâncias dos parâmetros. Além disso, embora seja afirmado na literatura que um intervalo PQ curto (<120 ms) devido ao encurtamento da duração da onda P é um sinal de alerta para o diagnóstico suspeito de DF, especialmente nos estágios iniciais, foi afirmado que o excesso de volume do AE é um fator de confusão para esta variável. Afirmou-se que a sensibilidade e a especificidade da variável podem mudar com a progressão da doença. Neste estudo, além da deficiência enzimática e mutação genética no sexo masculino, valores enzimáticos médios ou baixos no sexo feminino, positividade para mutação genética em membro da família, ou pelo menos um dos sinais/sintomas clássicos de DF ou O acúmulo de Gb3 foi necessário para o diagnóstico de DF clássica. ^38 , 39^ Essa situação mostra diferenças parciais com a metodologia do nosso estudo e pode ter feito com que a diferença nos achados relevantes do ECG não fosse observada em nosso estudo.

Assim como Sacchari et al. no estudo de *strain* , em nosso estudo, pacientes com CMH apresentaram maior volume de AE do que DF (Estudo de Sacchari et al.: 48,16 ± 14,3 mL/m ^2^ vs. 38,90 ± 14,9 mL/m ^2^ , respectivamente; p<0,001/Nosso estudo: 62,7±28,7 mL /m ^2^ vs. 53,5±11,3 mL/m ^2^ , respectivamente; p=0,028). Em Sacchari et al., ambos os grupos apresentaram menor deformação atrial e maior IMVE do que o controle, mas CMH e DF não diferiram. ^22^ Diferentemente dos estudos mencionados, o IMVE foi consideravelmente maior na CMH do que no DF em nosso estudo. Em Junguá et al., fração de ejeção do VE (FEVE) (69% vs. 65%, respectivamente; p=0,01), espessura miocárdica máxima (EMM) (21,8±4,8 mm vs. 16,2±3,5 mm, respectivamente; p<0,001), OVSVE (25% vs. 5%, respectivamente; p<0,001), o movimento anterior sistólico (MAS) (25% vs. 6,6%, respectivamente; p=0,01) foi considerado alto a favor da CMH. Entretanto, hipertrofia ventricular direita (23% vs. 3,4%, respectivamente; p=0,004), sVd (20,5±3,9 mm/m ^2^ vs. 18±2,5 mm/m ^2^ , respectivamente; p<0,001) e diâmetro tubular aórtico (18,4 ±3 mm/m ^2^ vs. 16,8±2,7 mm/m ^2^ , respectivamente; p=0,007) foram significativamente maiores em favor do DF. Na análise multivariada, o EMM é um preditor independente de CMH (p<0,001), e o sVd é um preditor independente de DF (p<0,01). ^5^ Em nosso estudo, IVSd (semelhante a Smid BE et al.), PWd, lateral e valores de E septal, PABs, IM moderada a grave, ERP (parâmetros recentemente analisados diferem dos estudos mencionados anteriormente), volume do AE e IMVE também foram maiores na CMH do que na DF. ^38^ Devido à regressão logística univariada, IVSd, PWd, IM moderada-grave e IMVE também são preditores de CMH. O DDVE grau I foi maior no DF, enquanto o DDVE grau III foi maior na CMH, mas não houve diferença em relação à FEVE. O sVd e o diâmetro da raiz aórtica não foram significativamente diferentes entre os grupos em nosso estudo. Os parâmetros OVSVE e MAS não foram verificados no DF, portanto não foi possível fazer comparações.

As diferenças populacionais e o estágio de progressão das doenças podem ter causado essas diferenças. Além disso, os pacientes foram internados no ambulatório dentro de um intervalo de tempo no estudo LVH-TR, e esses grupos de pacientes foram alcançados por triagem, e não por telefone. ^25^ Portanto, conhecer as diferenças durante a triagem de DF e CMH, possivelmente nos estágios iniciais, ajudará a diagnosticar ambas as doenças e a alcançar o tratamento rapidamente.

### Limitações

Embora nosso estudo seja multicêntrico nacional, ele só poderia ser realizado com um número limitado de pacientes porque as doenças relacionadas são raras e difíceis de diagnosticar. Como nosso estudo foi multicêntrico e avaliado com diferentes aparelhos de ecocardiografia por médicos de 22 centros diferentes, algumas diferenças podem ter sido observadas, embora o exame tenha sido realizado de acordo com as recomendações das diretrizes. Além disso, a exclusão de pacientes com bloqueio AV, BRD, FA e ritmo estimulado pode ter afetado outras características além do ECG entre os grupos. Devido à natureza retrospectiva do nosso estudo, alguns parâmetros não puderam ser comparados de forma tão semelhante como nos estudos, como dados de ecocardiografia de tensão e análise de testes genéticos para pacientes com CMH.

## Conclusão

Nosso estudo comparou as características demográficas, clínicas, uso de medicamentos, características bioquímicas desses dois grupos de doenças e dados detalhados de ECG e ECO com parâmetros recentemente examinados e mostrou que alguns parâmetros específicos poderiam auxiliar na diferenciação e diagnóstico precoce de CMH e DF. No futuro, um sistema de escore poderá ser criado planejando uma base de dados com estudos multinacionais para distinguir ambas as doenças, especialmente nas fases iniciais. Por fim, também podem ser criados documentos de consenso relacionados ao assunto. Portanto, nosso estudo pode orientar estudos futuros.
