# Supplementary material for: Diferenças entre Duas Condições Cardíacas Hipertróficas Distintas: Doença de Fabry Versus Cardiomiopatia Hipertrófica
Source: Arq Bras Cardiol. 2024 Jan 24;121(1):e20230229. [Article in Portuguese] doi: 10.36660/abc.20230229 (PMC11098584; doi:10.36660/abc.20230229)
Supplement: Translation

# Translation: Differences Between Two Distinct Hypertrophic Cardiac Conditions: Fabry Disease versus Hypertrophic Cardiomyopathy

**Authors:** Onur Akhan, Mehmet Kış, Tuncay Güzel, Mehdi Zoghi

**Affiliations:** 1 Turkey Bilecik Training and Reseach Hospital – Cardiology, Bilecik – Turkey; 2 Turkey Dokuz Eylul University Faculty of Medicine – Cardiology, Izmir – Turkey; 3 Turkey Health Science University, Gazi Yasargil Training and Research Hospital – Cardiology, Diyarbakır – Turkey; 4 Turkey Ege University Faculty of Medicine – Cardiology, Izmir – Turkey

**Keywords:** Cardiomyopathies, Left Ventricular Hypertrophy, Hypertrophic Cardiomyopathy, Fabry Disease

## Abstract

**Background:**

Hypertrophic cardiomyopathy (HCM) and Fabry disease (FD) are genetically inherited diseases with left ventricular hypertrophy (LVH) phenotype characteristics that cause adverse cardiac outcomes.

**Objectives:**

To investigate the demographic, clinical, biochemical, electrocardiographic (ECG), and echocardiographic (ECHO) differences between HCM and FD.

**Methods:**

60 HCM and 40 FD patients were analyzed retrospectively as a subanalysis of the ‘LVH-TR study’ after excluding patients with atrial fibrillation, pace rhythm, bundle branch blocks, and second and third-degree atrioventricular (AV) blocks. The significance level was accepted as <0.05.

**Results:**

Male gender (p=0.048) and creatinine (p=0.010) are significantly higher in favor of FD; however, ST depression (p=0.028), QT duration (p=0.041), interventricular septum thickness (IVSd) (p=0.003), posterior wall thickness (PWd) (p=0.009), moderate-severe mitral regurgitation (MR) (p=0.013), and LV mass index (LVMI) (p=0.041) are significantly higher in favor of HCM in the univariate analyses. In multivariate analysis, statistical significance only continues in creatinine (p=0.018) and QT duration (0.045). FD was positively correlated with creatinine (rho=0.287, p=0.004) and HCM was positively correlated with PWd (rho=0.306, p=0.002), IVSd (rho=0.395, p<0.001), moderate-severe MR (rho=0.276, p<0.005), LVMI (rho=0.300, p=0.002), relative wall thickness (RWT) (rho=0.271, p=0.006), QT duration (rho=0.213, p=0.034) and ST depression (rho=0.222, p=0.026).

**Conclusion:**

Specific biochemical, ECG, and ECHO characteristics can aid in the differentiation and early diagnosis of HCM and FD.

## Introduction

Fabry Disease (FD) is an X-linked lysosomal storage disease characterized by a-galactosidase A (a-Gal A) enzyme deficiency, which deposits globotriaosylceramide (Gb3) and related glycosphingolipids and affects the vascular, renal, neurologic, and cardiac systems. ^1 - 3^ FD can be classified as classical (severe) and late-onset (nonclassical - limited involvement of the organs). Cardiac involvement is the main prognostic factor and is often characterized by increased left ventricular (LV) wall thickness/mass, functional abnormalities, valvular heart disease, arrhythmias, and heart failure. ^3 , 4^ FD incidence ranges from 1/40,000 to 1/117,000, and the prevalence of FD in unexplained LV hypertrophy (LVH) ranges from 0% to 12%. ^1 , 5 - 10^ In a study in 2023, 19.5% of the patients with LV hypertrophy of unknown origin had decreased a-Gal A enzyme activity. ^11^ Owing to random X-chromosomal inactivation, female patients may exhibit as severely as male patients or be asymptomatic. ^1 - 4 , 12^ The FD’s X-linked nature causes diagnostic disparities between genders. Lower a-Gal activity evaluation in male patients is diagnostic. A-Gal A activity can be borderline or normal in females, so gene sequencing is sometimes the only way to diagnose. ^12 , 13^

HCM, an autosomal dominantly transmitted genetic heart disease (caused by a mutation in sarcomere proteins) with phenotypic LVH characteristics by an incidence of 1/500, causes severe cardiac consequences like ventricular arrhythmias, higher risk for heart failure, and sudden cardiac death. ^13 - 22^

HCM guidelines recommend investigating atypical causes of LVH, such as FD. ^5 , 15 , 16^ Women are underdiagnosed in both diseases, maybe due to disease characteristics or screening methods. ^12 , 17 , 18 , 23^ Early recognition and differentiation are needed to treat FD and HCM, especially cardiac involvement. Consequently, these disorders can be treated early, improving patients’ quality of life. ^3 , 4 , 15 , 16 , 20 - 24^

In our study, we aimed to evaluate the electrocardiographic (ECG) and echocardiographic (ECHO) features in a standardized way by minimizing the possible confounding factors with the exclusion of atrial fibrillation (AF), right bundle branch block (BBB), 2nd and 3rd degree atrioventricular (AV) blocks and the pace rhythm for the early diagnosis and differentiation of both diseases.

## Methods

Our study is a subgroup analysis of the national, multicenter, observational, screening ‘LVH-TR’ Study carried out in 22 centers between February 2020 and August 2021. In the LVH-TR study, the rate of patients diagnosed with HCM was 7.5% (66 patients). In the initial assessment in the LVH-TR study, factors such as hypertension, heart valve disorders, congenital heart conditions, chronic renal failure, infiltrative cardiomyopathies, athlete’s heart, and LV non-compaction cardiomyopathy were recognized as potential causes of left ventricular hypertrophy (LVH). The patients who were first evaluated and found to have LVH of unknown origin were subjected to the FD algorithm. Patients suspected of FD, with clinical (neuropathic pain, stomach ache, diarrhea, hypohidrosis, …), physical examination (angiokeratoma, hearing loss, corneal opacities…), laboratory (proteinuria…), ECG, ECHO, and cardiac magnetic resonance imaging findings were considered for further evaluation. Low α-Gal-A enzyme levels were detected in 43 LVH patients evaluated in our study’s FD group. Fabry patients in our study consisted of generally milder phenotypes. GLA gene mutation was generally seen as a missense mutation, and it was positive for 14 patients, of which 5 were female and 9 were male. Female patients did not have variant mutations of uncertain significance. ^25 , 26^ FD diagnosed with lower a-Gal activity in males and gene mutation analysis in females in patients with unexplained LVH. Although lower A-Gal A activity in men is sufficient for diagnosis, genetic analysis was performed for further evaluation. Notably, individuals having a preexisting diagnosis of FD were not included. Hence, it is seen that none of the patients identified with FD are administered enzyme replacement therapy (ERT) during the diagnostic period. Our study observed no familiar relationship among the patients diagnosed with FD.

The patients with 2nd and 3rd-degree AV blocks, BBB, AF, and pace rhythm were excluded from the study to compare ECG parameters in a standardized way. After the exclusion, 60 HCM and 40 FD patients in sinus rhythm were included in the study (Consort diagram and the summary of the results are shown in Central Figure).

Demographic characteristics, symptoms, medications, standard biochemistry measurement data (Blood glucose, HgA1c, hemoglobin, urea, creatine, glomerular filtration rate (GFR), high sensitive troponin T (TnT), Nt-pro-BNP), ECG and ECHO data were compared for the HCM and FD.

### Electrocardiographic analysis

At rest, heart rate, PR time, QRS width, and QT duration were evaluated from the standard 12-lead recording ECGs (10 mV/mm and 25 mm/s paper speed). The Bazett formula (QT/√RR) measures the corrected QT (QTc) interval. In addition, T negativity, T wave flattening, ST segment depression (the J-point depression threshold values are -0.05 mV in leads V2 and V3 and -0.1 mV in all other leads for both males and females), and Sokolow-Lyon Index (SLI = SV1 + RV5 or V6 ≥ 35 mm) positivity were recorded in line with the recommendations of the American Heart Association electrocardiography guidelines.

### Echocardiographic analysis

The standard transthoracic ECHO evaluation with the parasternal long axis, parasternal short axis, apical two-chamber, and apical four-chamber views are evaluated according to current guidelines. LVH is diagnosed with left ventricular mass index (LVMI), corrected for body surface area (BSA). LV mass is calculated with the formula 0.8×1.04×[(LVEDD + IVSd +PWd) ^3^ – LVEDD ^3^ ]+0.6, and LVMI is also calculated with the formula LV Mass / BSA. Relative wall thickness (RWT) is calculated by the formula (2 x LV-PWd / LVEDD), and M mode is used for the IVSd measurement in the diastolic phase (LVEDD: Left ventricular end-diastolic diameter, IVSd: Diastolic interventricular septum thickness, PWd: Diastolic posterior wall thickness).

After the exclusion of systemic diseases such as mitochondrial myopathies, glycogen/lysosomal storage diseases in children, FD, amyloidosis, sarcoid, hemochromatosis, and Danon cardiomyopathy and secondary causes of LVH such as athletes heart, hypertensive cardiomyopathy, chronic kidney disease (CKD), hemodynamic obstruction caused by left-sided obstructive lesions (valvular or subvalvular stenosis), or obstruction after antero-apical infarction and stress cardiomyopathy, wall thickness ≥15 mm in one or more myocardial segments in the left ventricle is diagnosed as HCM.

### α-Galactosidase a enzyme activity test

A fluorimetric method is used to measure the α-Gal-A (AGAL) enzyme activity, with dry blood testing obtained by aspirating peripheral venous blood samples onto dry blood sample paper (Substrate: 4-Methylumbelliferyl-α-D-galactopyranoside (TRC, M334475) - Inhibitor: N-Acetyl-D-galactosamine (Sigma, A2795)). Incubation was made at 37°C for 17 hours, and the reaction was stopped. Fluorescence was recorded in the fluorimeter (Ex: 366 nm - Em: 442 nm), and the calibration curve was created (4-Methylumbelliferone (Sigma M1381)). The threshold value was determined as >2.50 nmol/mL/hr for the usual range of AGAL activity by the receiver operating characteristic (ROC) test performed by the ‘Duzen laboratory group.’

### Mutation analysis

Regarding genotype analysis for FD, GLA gene sequence analysis was carried out with the next-generation sequencing (NGS) method. PCR products amplified from the isolated DNA were sequenced and compared to the reference sequence (NCBI Genomic Reference Sequence: NG 007119.1, NM 000169.2). The coding sequence mutations were reported among the mutations found in this database. Furthermore, the association of reported mutations with FD from the ‘HGMD’ and ‘ClinVar’ databases was added. Model analysis programs such as SIFT, Mutation t@ster, and PolyPhen-2 predictions have been added for mutations not in the database.

### Statistical analysis

IBM SPSS Statistics 25.0 Program was used. The conformity of numerical variables to the normal distribution was examined using the Kolmogorov-Smirnov test. To compare the two groups in terms of numerical variables, the independent samples t-test was used if the normal distribution was achieved, and the Mann-Whitney U test was used if not. Categorical variables were shown as numbers (n) and ratios (%). The relationship between categorical variables was examined with Pearson’s Chi-square and Fisher Exact test. The power of Hypertrophic Cardiomyopathy and FD value in predicting was evaluated with univariate and multivariate logistic regression analyses. Odd’s Ratio (OR) and 95% CI values were recorded. Parameters predicting hypertrophic cardiomyopathy and FD were evaluated with Spearman correlation analysis. Rho and p values were recorded. Descriptive data were expressed as mean ± standard deviation (SD) values for normally distributed continuous variables and median (interquartile range - IQR) values for non-normally distributed variables. The significance level for all hypotheses was accepted as <0.05.

## Results

In both diseases, the proportion of male patients was higher. Palpitation, dizziness symptoms, and the rate of beta-blocker use were encountered at a higher rate in HCM than in FD. GFR, Creatinine, and TnT were significantly higher in favor of FD. Comparisons between groups regarding demographic, clinical characteristics, drug use, and biochemistry parameters are summarized in Table 1 .

**Table 1:** – Comparison of Demographic, Clinical Characteristics, Drug Usage, and Biochemical Parameters

Parameters	HCM (n=60)	Fabry (n=40)	p-value
Age, mean±std	53.9±14.0	51.9±12.9	0.483
Male gender, n (%)	42 (70.0)	35 (87.5)	0.042
Body Mass Index, kg/m ^2^ , mean±std	29.0±5.0	27.5±4.2	0.139
Body Surface Area, mean±std	1.94±0.19	1.96±0.17	0.655
Systolic Blood Pressure, mean±std	132+22	138±23	0.208
Diastolic Blood Pressure, mean±std	79±11	84±13	0.055
Hyperlipidemia, n (%)	13 (21.7)	5 (12.5)	0.242
Diabetes Mellitus, n (%)	10 (16.7)	5 (12.5)	0.568
**Symptoms**			
Chest pain, n (%)	27 (45.0)	16 (40.0)	0.621
Palpitations, n (%)	33 (55.0)	9 (22.5)	0.001
Dyspnea, n (%)	34 (56.7)	16 (40.0)	0.102
Tiredness, n (%)	30 (50.0)	18 (45.0)	0.624
Dizziness, n(%)	21 (35.0)	6 (15.0)	0.027
Syncope, n (%)	6 (10.0)	2 (5.0)	0.367
**Drug Usage**			
Calcium channel blocker, n (%)	12 (20.0)	10 (25.0)	0.554
β- blocker, n (%)	51 (85.0)	20 (50.0)	<0.001
Amiodarone, n (%)	2 (3.3)	0	0.515*
Statin, n (%)	11 (18.3)	5 (12.5)	0.436
**Biochemical Parameters**			
Hb, g/dl, mean±std	13.8±1.6	13.8±2.0	0.852
HbA1c, %, mean±std	5.7±0.7	5.8±0.5	0.705
Creatinine, mg/dl, median (interquartile range)	0.90 (0.80-1.00)	1.00 (0.86-1.34)	0.004
GFR (ml/min)	90.7±27.1	73.9±31.0	0.005
Nt±probnp, pg/ml, median (interquartile range)	321 (212-481)	192 (156-656)	0.240
Troponin, pg/ml, median (interquartile range)	11.5 (5.0-22.7)	44.0 (15.2-125.0)	<0.001

HCM: hypertrophic cardiomyopathy; CCB: calcium channel blocker; BB: Beta blocker; Hb: hemoglobin; GFR: glomerular filtration rate; *Fischer-Exact Test.

The differences detected in ECG features were that ST depression was higher, and the QT duration was longer in HCM than in FD *.* Left ventricular diastolic dysfunction (LVDD) grade I was higher in favor of FD, while LVDD grade III was higher in favor of HCM. Table 2 shows all ECG and ECHO parameters for other specificities and differences. In our study, we also have the information that the percentage of LVOTO in HCM patients was 38.3%, and the percentage of SAM was 43.3%.

**Table 2:** – Comparison Of Electrocardiography and Echocardiography Parameters

Electrocardiography Parameters		HCM (n=60)	Fabry (n=40)	p-value
Heart rate, mean±std		73±15	73±14	0.883
1st degree AV block, n (%)		8 (13.3)	6 (15.0)	0.814
T negativity, n (%)		39 (65.0)	22 (55.0)	0.315
T flattening, n (%)		16 (26.7)	9 (22.5)	0.637
ST depression, n (%)		39 (65.0)	17 (42.5)	0.026
PR distance, msn, mean±std		162.3±31.8	151.5±36.7	0.124
QRS duration, msn, mean±std		102.3±12.3	98.0±13.9	0.106
QT duration, msn, mean±std		412.7±65.1	388.1±39.1	0.035
QTc duration, mean±std		447.3±70.9	423.7±39.1	0.058
Sokolow-Lyon Indeks, n (%)		44 (73.3)	24 (60.0)	0.161
**Echocardiography Parameters**				
LVEF, mean±std		58.6±7.4	57.9±5.8	0.588
RVEF ≥ 45, n (%)		57 (95.0)	40 (100.0)	0.151
LVDD Classification	LVDD Grade I	25 (41.7)	24 (60.0)	0.016
	LVDD Grade II	18 (30.0)	8 (20.0)	
	LVDD Grade III	14 (23.3)	2 (5.0)	
LVEDD, mm, mean±std		43.2±6.2	44.7±3.7	0.148
LVESD, mm, mean±std		28.8±7.3	27.7±4.0	0.310
IVSd, mm, mean±std		19.5±4.4	16.5±4.1	0.001
PWd, mm, mean±std		15.3±4.0	13.3±2.5	0.002
Lateral E, mm, mean±std		8.9±2.9	6.7±1.6	<0.001
Septal E, mm, mean±std		6.3±2.3	5.5±1.6	0.041
Aortic Root, mm, mean±std		27.1±4.5	27.0±4.3	0.898
Sinus Valsalva, mm, mean±std		35.4±4.0	35.5±4.7	0.917
Ascending Aorta, mm, mean±std		34.9±3.7	35.1±3.9	0.848
RV Diameter, mm, mean±std		28.9±4.7	27.6±3.9	0.154
TAPSE mm		20.7±3.5	19.7±4.0	0.232
PABs, mmHg		29.8±9.6	23.9±5.6	<0.001
E/e′ mean > 14, n (%)		23 (38.3)	18 (45.0)	0.507
LA Volume, mL/m ^2^ , mean±std		62.7±28.7	53.5±11.3	0.028
Peak TR velocity > 2.8 m/s, n (%)		13 (21.7)	7 (17.5)	0.610
Moderate-Severe MR, n (%)		16 (26.7)	2 (5.0)	0.006
LVMI, g/m ^2^ , mean±std		161.5±32.0	143.7±50.1	0.033
RWT, mean±std		0.72±0.25	0.60±0.15	0.003

*HCM: hypertrophic cardiomyopathy; AV: atrioventrıcular; LVEF: left ventricular ejection fraction; RVEF: right ventricular ejection fraction; LVEDD: left ventricular end diastolic diameter; LVESD: left ventricular end systolic diameter; IVSd: diastolic ınterventricular septum thickness; PWd: diastolic posterior wall thickness; PABs: systolic pulmonary artery pressure; LA: left atrium; TR: tricuspid regurgitation; MR: mitral regurgitation, AR: aortic regurgitation, LVMI: left ventrıcular mass ındex; LV: left ventricle; RWT: relative wall thickness; SAM: systolic anterior motion.*

Predictors and independent predictors were summarized in Table 3 due to the univariate and multivariate logistic regression analysis. These results will also be further explained in the discussion section. In Spearman’s correlation analysis, we also found a moderate positive correlation between FD and creatinine and a moderate positive correlation between HCM and PWd, IVSd, moderate-severe MR, LVMI, RWT, and a weak positive correlation between HCM and QT duration and ST depression ( Table 4 ).

**Table 3:** – Univariate - Multivariate Regression Analyses for Determining Predictor of Hypertrophic Cardiomyopathy and Fabry Disease

Parameters	Univariate Analysis		Multivariate Analysis	
	OR (95% CI)	p-value	OR (95% CI)	p-value
Male gender	0.333 (0.112-0.989)	0.048	0.561 (0.154-2.046)	0.381
Creatinine	7.313 (1.623-32.954)	0.010	6.405 (1.371-29.925)	0.018
Troponin	1.004 (0.999-1.010)	0.130		
ST depression	2.513 (1.105-5.712)	0.028	2.025 (0.719-5.702)	0.181
QT duration	0.991 (0.983-1.000)	0.041	0.989 (0.978-1.000)	0.045
IVSd	0.836 (0.744-0.940)	0.003	0.945 (0.796-1.121)	0.513
PWd	0.820 (0.707-0.951)	0.009	0.847 (0.695-1.031)	0.098
LA Volume	0.982 (0.963-1.001)	0.063		
Moderate-Severe MR	6.909 (1.492-31.994)	0.013	4.660 (0.702-30.939)	0.111
LVMI	0.987 (0.975-0.999)	0.041	1.004 (0.985-1.023)	0.692

*OR: odds ratio; CI: confidence ınterval; IVSd: diastolic ınterventricular septum thickness ; PWd: diastolic posterior wall thickness; MR: mitral regurgitation; LVMI: left ventricular mass ındex; RWT: relative wall thickness.*

**Table 4:** – Spearman’s Correlation Analysis for Determine Predictor of Hypertrophic Cardiomyopathy and Fabry Disease

	Creatinine	ST Depr.	QT Dur.	IVSd	PWd	Mod.-Sev. MR	LVMI	RWT
**rho**	0.287	-0.222	-0.213	-0.395	-0.306	-0.276	-0.300	-0.273
p-value	0.004	0.026	0.034	<0.001	0.002	0.005	0.002	0.006

*Depr.: depression; Dur.: duration; IVSd: diastolic ınterventricular septum thickness; PWd: diastolic posterior wall thickness; Mod.-Sev: moderate-severe; MR: mitral regurgitation; LVMI: left ventricular mass ındex; RWT: relative wall thickness. + values are correlated with Fabry Disease / - values are correlated with Hypertrophic Cardiomyopathy.*

## Discussion

Our study examines FD and HCM demographic, clinical, biochemical, ECG, and ECHO features. Due to their similarities and potential adverse outcomes, these two diseases must be distinguished. Like the literature, our study population had more male patients in both diseases. ^1 - 4 , 15 - 18^ The hereditary traits of FD, its asymptomatic course in females, possible screening bias, and genetic and hormonal modifiers in HCM explain this situation. In our study, the male sex percentage was also significantly higher in FD than in HCM. However, Jungua et al. did not detect a significant difference in male gender (p=0.42), and in the study of Sacchari et al., no p-value was given regarding male gender discrepancy. ^5 , 22^ Possible variances in demographic characteristics may have caused this difference.

Although there are some differences in the order of frequency, angina, dyspnea, palpitations, and syncope are the common cardiac symptoms in both diseases. ^15 , 16 , 24^ Syncope is also included in HCM’s sudden cardiac death risk scoring. Screening examinations rather than symptoms lead to HCM diagnosis, according to guidelines. ^15 , 16^ Although the common symptoms in our study were consistent with the guidelines, the differences between the groups were considered coincidental because these symptoms were subjective and nonspecific.

Beta-blockers, verapamil, and diltiazem lower heart rate to lower LV diastolic pressures and improve LV filling in HCM. ß-blockers are initially titrated to the maximally tolerable dose for symptomatic left ventricular outflow tract obstruction (LVOTO). ^15 , 16^ In addition to enzyme replacement and chaperone therapy, concomitant medications can also be used for the reduction of complaints, delaying/preventing the progression of organ manifestations, and improving quality of life in FD. ^27 , 28^ In our study, ß-blocker usage was significantly higher in HCM compared to FD, similar to the Jungua N. et al. study (84% vs. 26%, respectively; p<0.001). ^5^

The gradual accumulation of GL3 in all types of renal cells, predominantly in podocytes, and the release of inflammatory mediators causes FD nephropathy. ^29^ CKD is excluded from the HCM due to the definition in the current guidelines. ^15^ But HCM can also impair renal function secondary to adverse cardiac outcomes. ^30^ In our study, creatinine and TnT are significantly higher, and GFR is significantly lower in FD than in HCM *(all p values <0.05)* . Since the LVH-TR study was a screening study, and first of all, the conditions with a clear LVH etiology were determined and further evaluated for the unexplained LVH etiology, and then the diagnosis of FD was reached, and perhaps due to population characteristics, renal involvement in FD patients may have been low levels (GFR: 73.9±31.0 ml/min in FD). ^25^ FD and HCM can raise myocardial injury indicators like troponin (especially with LVOTO). ^31 - 33^ It has been stated that endothelial dysfunction may be observed in Fabry cardiomyopathy (defined as LV wall thickness of 12 mm and above in Cardiac MRI imaging) due to changes in angiogenesis markers. In this study, GFR values were 95.7 ± 19.6 (ml/min/1.73 m ^2^ ) in the group without cardiomyopathy, 71.9 ± 21.5 in the group with cardiomyopathy, and 3.7 ± 0.8 and 28.8 ± 25.2 in TnT (pg/ml), respectively. ^31^ In a study on serum biomarkers in HCM patients with preserved LVEF, the decrease in strain parameters was shown as the reason for the increase in TnT values against the healthy control group. In this study, the median troponin T value was 14.25 pg/ml (IQR: 9.98, 22.83) in 64 HCM patients, 65% of whom had LVOTO and 10% who had AF. ^34^ The increase in TnT values in HCM patients has been associated with arrhythmias and disease stages. ^35^ In our study, TnT values were similar to those mentioned above. Differences between the groups can be interpreted with HCM with LVOTO ratio (38%), exclusion of arrhythmias, and possible confounders such as differences related to renal function parameters.

In Jungua et al. study, the right BBB (54% vs. 22%, respectively; p=0.001), QRS duration (117±27 msn vs. 99±25, respectively; p<0.001), and SLP (p=0.004) were significantly higher in the FD group than in HCM. However, no significant difference was found in other parameters (all p values<0.05), such as QTc (p=0.58). ^5^ In our study, HCM had higher QT duration and ST depression (measured not seen in previous studies) than FD. SLI positive was comparable in both groups (p=0.161). In univariate analysis, ST depression and QT duration predict HCM; however, only QT duration predicts HCM in multivariate analysis. ST depression, QT duration, and HCM were weakly positively correlated in Spearman’s correlation analysis. As HCM and FD progress, QT may prolong. ^36 , 37^ Hence, the HCM LVOTO percentage and FD in earlier stages may have affected the parameter discrepancies. In addition, although it is stated in the literature that a short PQ interval (<120 ms) due to shorting of P wave duration is a red flag for the suspect diagnosis of FD, especially in the early stages, it has been stated that excess LA volume is a confounding factor for this variable. It has been stated that the variable’s sensitivity and specificity may change with disease progression. In this study, in addition to enzyme deficiency and genetic mutation in the male gender, average or low enzyme values in the female gender, genetic mutation positivity in a family member, or at least one of the classic signs/symptoms of FD or Gb3 accumulation were required for the diagnosis of classic FD. ^38 , 39^ This situation shows partial differences with the methodology of our study and may have caused the difference in the relevant ECG findings to be not observed in our study.

Like Sacchari et al. strain study, in our study, HCM patients had larger LA volume than FD (Sacchari et al. study: 48.16 ± 14.3 mL/m ^2^ vs. 38.90 ± 14.9 mL/m ^2^ , respectively; p<0.001 / Our study: 62.7±28.7 mL/m ^2^ vs. 53.5±11.3 mL/m ^2^ , respectively; p=0.028). In Sacchari et al. study, both groups had lower atrial strain and higher LVMI than the control, but HCM and FD did not differ. ^22^ Unlike the mentioned studies, LVMI was considerably higher in HCM than FD in our study. In Jungua et al. study, LV ejection fraction (LVEF) (69% vs. 65%, respectively; p=0.01), maximal myocardial thickness (MMT) (21.8±4.8 mm vs. 16.2±3.5 mm, respectively; p<0.001), LVOTO (25% vs. 5%, respectively; p<0.001), systolic anterior motion (SAM) (25% vs. 6.6%, respectively; p=0.01) were found to be high in favor of HCM. However, right ventricular hypertrophy (23% vs. 3.4%, respectively; p=0.004), sVd (20.5±3.9 mm/m ^2^ vs. 18±2.5 mm/m ^2^ , respectively; p<0.001), and tubular aortic diameter (18.4±3 mm/m ^2^ vs. 16.8±2.7 mm/m ^2^ , respectively; p=0.007) were significantly higher in favor of FD. In multivariate analysis, MMT is an independent predictor of HCM (p<0.001), and sVd is an independent predictor of FD (p<0.01). ^5^ In our study, IVSd (similar to Smid BE et al.), PWd, lateral and septal E values, PABs, moderate-severe MR, RWT (newly analyzed parameters differ from previously mentioned studies), LA volume, and LVMI were also higher in HCM than in FD. ^38^ Due to univariate logistic regression, IVSd, PWd, moderate-severe MR, and LVMI are also predictors of HCM. LVDD grade I was higher in FD, while LVDD grade III was higher in HCM, but there was no difference regarding LVEF. sVd and aortic root diameter were not significantly different between groups in our study. LVOTO and SAM parameters were not checked in FD, so comparisons could not be made.

Population differences and the stage of the progression of the diseases may have caused these differences. In addition, the patients were admitted to the outpatient clinic within a time interval in the LVH-TR study, and these patient groups were reached due to screening, not by calling. ^25^ Therefore, knowing the differences during FD and HCM screening, possibly in earlier stages, will help diagnose both diseases and reach the treatment quickly.

### Limitations

Although our study is a national multicenter study, it could only be done with a limited number of patients because the related diseases are rare and difficult to diagnose. Since our study was multicenter and evaluated with different echocardiography devices by physicians from 22 different centers, some differences may have been observed even though the examination was carried out in line with the guideline recommendations. In addition, excluding patients with AV block, BBB, AF, and paced rhythm may have affected other characteristics rather than ECG between groups. Due to the retrospective nature of our study, some parameters could not be compared as similarly as in the studies like strain echocardiography data and genetic test analysis for HCM patients.

## Conclusion

Our study compared these two disease groups’ demographic, clinical features, drug use, biochemical characteristics, and detailed ECG and ECHO data with newly examined parameters and showed that some specific parameters could aid in the differentiation and early diagnosis of HCM and FD. In the future, a scoring system can be created by planning a database with multinational studies to distinguish both diseases, especially in the early stages. Finally, consensus documents related to the subject can also be created. Therefore, our study can guide future studies.

**Central Illustration:** : Differences Between Two Distinct Hypertrophic Cardiac Conditions: Fabry Disease versus Hypertrophic Cardiomyopathy Consort diagram and summary of the results.
